# TKTL1 expression in human malign and benign cell lines

**DOI:** 10.1186/1471-2407-15-2

**Published:** 2015-06-10

**Authors:** Ulrike Kämmerer, Olivier Gires, Nadja Pfetzer, Armin Wiegering, Rainer Johannes Klement, Christoph Otto

**Affiliations:** Department of Obstetrics and Gynaecology, University of Würzburg Hospital, Josef-Schneider-Str. 4, D-97080 Würzburg, Germany; Department of Otorhinolaryngology, Head and Neck Surgery, Ludwig-Maximilians-University, Marchioninistr. 15, D-81377 Munich, Germany; Apoptosis and Tumour Metabolism Lab, CRUK Beatson Institute, Garscube Estate, Switchback Road, Glasgow, G61 1BD United Kingdom; Department of General, Visceral, Vascular and Paediatric Surgery, University Hospital of Würzburg, Oberdürrbacher Str. 6, D-97080 Würzburg, Germany; Department of Radiotherapy and Radiation Oncology, Leopoldina Hospital Schweinfurt, Gustav-Adolf-Straße 8, D-97422 Schweinfurt, Germany; Experimental Surgery, Experimental Transplantation Immunology, Department of General, Visceral, Vascular and Paediatric Surgery, University Hospital of Würzburg, Oberdürrbacher Str. 6, D-97080 Würzburg, Germany

**Keywords:** Cancer cell lines, TKTL1, Immunohistochemistry, RT-qPCT

## Abstract

**Background:**

Overexpression of transketolase-like 1 protein TKTL1 in cancer cells has been reported to correlate with enhanced glycolysis and lactic acid production. Furthermore, enhanced TKTL1 expression was put into context with resistance to chemotherapy and ionizing radiation. Here, a panel of human malign and benign cells, which cover a broad range of chemotherapy and radiation resistance as well as reliance on glucose metabolism, was analyzed *in vitro* for TKTL1 expression.

**Methods:**

17 malign and three benign cell lines were characterized according to their expression of TKTL1 on the protein level with three commercially available anti-TKTL1 antibodies utilizing immunohistochemistry and Western blot, as well as on mRNA level with three published primer pairs for RT-qPCR. Furthermore, sensitivities to paclitaxel, cisplatin and ionizing radiation were assessed in cell survival assays. Glucose consumption and lactate production were quantified as surrogates for the “Warburg effect”.

**Results:**

Considerable amounts of tktl1 mRNA and TKTL1 protein were detected only upon stable transfection of the human embryonic kidney cell line HEK293 with an expression plasmid for human TKTL1. Beyond that, weak expression of endogenous tktl1 mRNA was measured in the cell lines JAR and U251. Western blot analysis of JAR and U251 cells did not detect TKTL1 at the expected size of 65 kDa with all three antibodies specific for TKTL1 protein and immunohistochemical staining was observed with antibody JFC12T10 only. All other cell lines tested here revealed expression of tktl1 mRNA below detection limits and were negative for TKTL1 protein. However, in all cell lines including TKTL1-negative HEK293-control cells, antibody JFC12T10 detected multiple proteins with different molecular weights. Importantly, JAR and U251 did neither demonstrate an outstanding production of lactic acid nor increased resistance against chemotherapeutics or to ionizing radiation, respectively.

**Conclusion:**

Using RT-qPCR and three different antibodies we observed only exceptional occurrence of TKTL1 in a panel of malignant human cell lines *in vitro*. The presence of TKTL1 was unrelated to either the rate of glucose consumption/lactic acid production or resistance against chemo- and radiotherapy.

**Electronic supplementary material:**

The online version of this article (doi:10.1186/1471-2407-15-2) contains supplementary material, which is available to authorized users.

## Background

Transketolase (TKT; EC: 2.2.1.1) is a thiamine-dependent key enzyme involved in the non-oxidative branch of the pentose phosphate pathway (PPP), which catalyzes the transfer of a 2-carbon fragment (H_2_C(OH)-CO-) from xylulose 5-phosphate to either ribose 5-phosphate (to form sedoheptulose 7-phosphate) or erythrose 4-phosphate (to form fructose 6-phosphate). TKT, together with the transaldolase enzyme, reversibly links the PPP to glycolysis [1, 2]. Both pathways occur exclusively in the cytosol and, depending on the metabolic demands of the cell, the PPP provides precursors for biosynthetic reactions or metabolites for glycolysis [3]. An altered PPP was reported to be involved in carcinogenesis and resistance to chemotherapeutic intervention, although the underlying mechanisms remain to be elucidated in more detail [4].

An alteration in the activity of the TKT enzyme was proposed to be responsible for the thiamine-deficiency related neurological disorder Wernicke-Korsakoff syndrome [5]. A genomic-wide screen revealed a new gene identified in embryonic brain and heart tissue, located on the Xq28 region next to genes of cancer/testis antigens (CTA). This gene was termed TKT-related (TKR) gene [6]. The TKR gene corresponds to an open reading frame of 540 codons with a nucleotide sequence demonstrating 60-67% homology to the highly conserved TKT gene of humans, mice and other species. TKR gene was assumed to be a pseudogene based on the presence of a premature stop-codon within the predicted open reading frame and a deletion of exon 3 of TKT, which encodes for amino acids important for the biochemical function of the TKT enzyme [7]. Later the TKR gene has been renamed transketolase-like 1 (TKTL1) gene; together with TKT and TKTL2, another TKT-like gene, it represents one of three isoforms of TKT [2, 7, 8]. Of note, the TKTL1 gene has been found especially in human testis tissue during germ cell maturation and in corresponding seminal plasma of fertile donors, a finding consistent with the CTA location of the gene [9, 10].

The commercially available monoclonal antibody JFC12T10 that recognizes the C-terminal fragment of recombinant TKTL1 protein [7] was used for immunohistochemistry and Western blot studies on a large panel of human tissues and cell lines to analyze TKTL1 expression [11–22]. The specific detection of TKTL1 protein in paraffin sections with JFC12T10 allowed the discrimination between healthy TKTL1-negative epithelium and TKTL1-positive carcinoma cells [7]. Mutations within the TKTL1 gene have been suggested to tissue-specific transcripts of different sizes encoding an enzymatically active transketolase protein as well as different smaller protein isoforms [7]. Western blot analyses of five human cancer cell lines demonstrated that JFC12T10 identifies different TKTL1 protein isoforms with molecular weights of 40 and 75 kDa, respectively [7], the calculated molecular weight of the original TKTL1 protein being 65.4 kDa.

Using immunohistochemistry, TKTL1 was found overexpressed in a variety of human cancer tissues, and a strong TKTL1 signal correlated with tumor invasiveness [23, 24]. Langbein and coworkers described that 9 out of 10 tumors found to be metastatic also showed a strong staining of TKTL1 using the JFC12T10 antibody [23]. In contrast to TKTL1, TKT and TKTL2 expression was not up-regulated in these tissues [23]. It was further proposed that tumors characterized by an up-regulation of TKTL1 demonstrated a TKTL1-based pathway with increased glucose metabolism and production of large amounts of lactic acid [7]. The enhanced glucose metabolism of solid malignant tumors with increased glucose consumption and excess amounts of lactic acid production, even in the presence of oxygen, is known as aerobic glycolysis, also termed “Warburg effect” [25, 26]. The widespread clinical application of the imaging technique positron-emission tomography (PET) using the radiolabeled glucose analogue FDG demonstrates such enhanced aerobic glycolysis in most tumors [27]. Constitutive up-regulation of glucose metabolism by tumor cells arises as an adaption to local hypoxia [27] and TKTL1 should enable cells to catabolize glucose in an oxygen-independent manner [7, 8]. Indeed, TKTL1 suppression in malignant cells resulted in significantly slower cell growth as well as reduced glucose consumption and lactic acid production [28]. TKTL1 overexpression in solid tumors was suggested to correlate with enhanced aerobic glycolysis [29] and resistance to chemotherapy and radiation [30]. However, the postulated enzymatic key role of TKTL1 for the energy metabolism of malignant cells has been a subject of recent controversy [30–34]. A protein sequence alignment of TKT and TKTL1 exhibited that TKTL1 lacks several important amino acid residues necessary for the enzymatic action of all TKT enzymes characterized so far [31]. From the results of the spatial structure of TKTL1 it was resumed that TKTL1 is unlikely to be a thiamine-dependent protein capable of catalyzing the TKT reaction [32]. Furthermore, two independent research groups were unable to detect any transketolase activity of a TKTL1 mimic protein. This was generated from TKT via deletion of the 38 amino acid residues from TKT, which are lacking in the original TKTL1 sequence [33, 34].

A positive staining of cancer tissues with the TKTL1-specific antibody clone JFC12T10 was reported to correlate with a poor patient outcome in some cancer entities including laryngeal squamous cell carcinoma, non small cell lung cancer, tumors of the ocular adnexa, papillary thyroid carcinoma and rectal cancer, indicating TKTL1 as a marker of prognostic relevance [15, 19, 23, 24, 35]. However, other studies failed to show a correlation between staining intensity for TKTL1 and clinical parameters [14, 36–38]. Hence, the inconsistency of published results raised concerns about the prognostic relevance of TKTL1. We therefore decided to analyze the expression of TKTL1 in a panel of 17 established malign cell lines and three benign control cell types (two primary and one established cell line) by immunohistochemistry, Western blot and quantitative PCR (RT-qPCR). Further, we characterized these cells by evaluating their survival after treatment with the chemotherapeutic drugs taxane and cisplatin, and ionizing radiation, respectively. In addition, glucose consumption and production of lactic acid were measured in all cell lines at 21% oxygen to address a potentially increased “Warburg effect” that may correlate with successful detection of TKTL1 expression by immunohistochemistry, Western blot and RT-qPCR. In order to compare results, immunohistochemistry and Western blot were performed with the monoclonal anti-TKTL1 antibody clones JFC12T10 and 1C10 as well as the polyclonal anti-TKTL1 antibody Sigma Prestige.

Our results indicate that TKTL1 expression is rare in cell lines tested and unrelated to both their rate of lactic acid production and resistance against chemo- and radiotherapy. Additionally, staining patterns of all three TKTL1-specific antibodies were not matched. The antibodies Sigma Prestige and 1C10 detected a single, distinct protein band of TKTL1 at the expected molecular weight in the TKTL1-transfected control cell line, and were negative in all remaining cell lines tested. The antibody clone JFC12T10 correctly detects the TKTL1 protein at the expected size of 65.4 kDa in these transfected control cells and additional multiple protein bands with different molecular weights, which were also visible in all other tested cell lines.

## Methods

### Cell lines

The cell lines investigated herein were directly obtained from the companies given in Table 1 to warrant their correct identity. For all experiments described, each cell line was routinely cultured in a 1:1 mixture of DMEM/Ham's F-12 supplemented with 10% (v/v) fetal calf serum (FCS) and 10 ng/ml gentamycine (all reagents from PAA, Coelbe, Germany) at 37°C in the presence of 5% CO_2_ and 21% oxygen***.*** Cells were cultured in 75 ml culture flasks (Biochrom, Berlin, Germany) as monolayers and harvested at 80-90% confluence using a cell-scraper (Biochrom) for further experiments. HEK293 cell transfectants stably producing full-length TKTL1 protein (293pCAG TKTL1) were used as positive control cells. HEK293 cells transfected with empty expression vector (293pCAG Δ) do not produce TKTL1 protein and were used as negative control cells. Both transfectants have been previously described in detail [21]. For the present study the transfectants were named as follows: 293pCAG TKTL1 = HEK293-TKTL1 transfectants and 293pCAG Δ = HEK293-control transfectants.

**Table 1:** **Cell lines and primary cells**

Name	Tissue type	Source	Literature
Fibroblasts	Uterine fibroblasts	PC	
HTC116	Colorectal carcinoma	DSMZ	[60, 61]
HEK 293- control	Embryonic kidney cells	DOO	[21]
HEK 293-TKTL1	Embryonic kidney cells	DOO	[21]
HeLa	Adenocarcinoma of the cervix	CLS	[62]
HepG2	Hepatocellular carcinoma	CLS	[63]
HT-29	Adenocarcinoma of the colon	CLS	[64]
HUVEC	Human umbilical vein endothelial cells	PC	
JAR	Choriocarcinoma	CLS	[65]
JEG	Choriocarcinoma	CLS	[66]
23132/87	Adenocarcinoma of the stomach	IOP	[67, 68]
MCF-7	Invasive breast ductal carcinoma	CLS	[69]
MDA-MB 231	Adenocarcinoma of the breast	CLS	[70]
Mel2a	Metastatic melanoma	DOD	[71]
OVCAR	Ovarian carcinoma	ATCC	[72]
PA1	Teratocarcinoma of the ovary	CLS	[73]
SiHa	Cervical carcinoma	ATCC	[74]
SKOV3	Adenocarcinoma of the ovary	CLS	[64]
U251, U87	Glioblastoma	CLS	[75]
WiDr	Adenocarcinoma of the colon	CLS	[76]
WS1	Embryo-derived skin fibroblasts	CLS	[77]

**ATCC:** American Type Culture Collection, Manassas, Virginia, USA (http://www.lgcstandards-atcc.org); **CLS:** Cell Lines Services GmbH, Eppelheim, Germany (http://www.cell-lines-service.de);

**DOD:** Department of Dermatology, University of Würzburg Hospital, Germany; **DOO:** Department of Otorhinolaryngology, Head and Neck Surgery, Ludwig-Maximilians-University, Munich, Germany; **DSMZ:** German Collection of Microorganisms and Cell Cultures, Leibniz Institute, Braunschweig, Germany (http://www.dsmz.de); **IOP:** Institute of Pathology, University of Würzburg, Germany.; **PC**: PromoCell GmbH, Heidelberg, Germany.

### Immunohistochemistry of formalin-fixed, paraffin-embedded cell pellets

Approximately 1x10^6^ cells per cell line were harvested upon trypsinization, washed twice with PBS and fixed for 1 h in 4% PBS-buffered formalin at room temperature. All centrifugation steps were performed at 280x g at 4°C for 10 min. The pellet was then re-suspended in 150 μl PBS and mixed with the same quantity of pre-cooled 2% high-melting agarose (Fermentas GmbH, St. Leon-Roth, Germany). The mixture was cooled down on ice. The resulting clot was first transferred to a sample-embedding capsule and then to a 4% PBS-buffered formalin solution. The dehydration with graded alcohols and xylene, and the embedding into paraffin (Histosec, Merck, Darmstadt, Germany) were done automatically in parallel to tissue biopsy samples at the Institute of Pathology, University of Würzburg, in a Leica ASP200 S embedding unit. The resulting paraffinized cell clot samples were then set into paraffin blocks. Paraffin blocks with cell samples were cut into 2 μm thick sections and mounted up on aminopropylethoxysilane (APES)-coated slides. Slides for immunohistochemistry were rehydrated in descending concentrations of ethanol before being heated for antigen unmasking in 10 mmol/l sodium citrate buffer (pH 6.0) in a microwave oven at 600 W for 5 min. After rinsing in distilled water, inhibition of endoperoxidase was performed by incubating sections for 10 min in 3% H_2_O_2_ in methanol. Slides were washed in PBS and incubated with 1% human immunoglobulin (Beriglobin, CSL Behring, Marburg, Germany) in PBS for 15 min to block FC-receptors [39]. Subsequently, slides were incubated with monoclonal mouse anti-TKTL1 antibody (clone JFC12T10, stock solution: 1 mg/ml; Linaris) or polyclonal anti-TKTL-1 antibody (Sigma prestige HPA000505, stock solution: 0.1 mg/ml; Sigma-Aldrich, Deisenhofen, Germany) diluted in antibody dilutent (DAKO, Hamburg, Germany). USB antibody used in Western blotting was not applicable for immunohistochemistry. Optimal antibody concentrations were determined in a series of dilutions with HEK293-TKTL1 transfectants and tumour tissue previously found to be TKTL1 positive [37, 40–43]. A dilution of 1:200 from the stock solution was optimal for JFC12T10 and 1:20 for the SigmaPrestige antibody. After 60 min of incubation at room temperature in a humidified chamber, slides were washed in PBS and incubated with the horseradish-labelled LSAB2 secondary antibody mixture (DAKO) according to the manufacturer’s protocol. Staining was developed by adding 3,3’diaminobenzidine (DAB ready to use, DAKO) with subsequent counterstaining using haematoxylin. Afterwards, sections were dehydrated by washing in graded ethanol and then embedded in Vitro-Clud (Langenbrink, Germany). To obtain a maximum of homogeneous results all staining procedures were carried out in parallel in one session and repeated twice. Stained cells were photographed at 40x magnification with a KEYENCE Biozero Microscope (Keyence Corporation, Osaka, Japan) applying Z-stack technology.

### Western blotting

**Figure 1:**
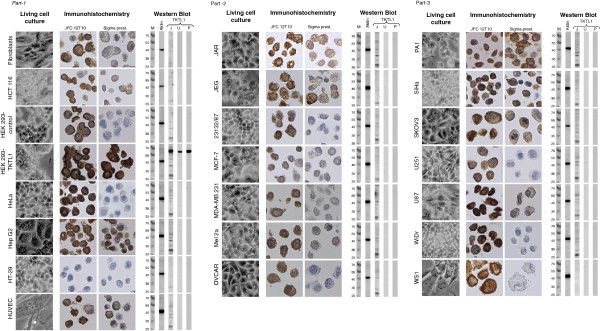
**Detection of TKTL1 expression on protein levels.** The cell morphology in cell culture is shown in the left column (light microscope, phase contrast, magnification x40 plus digital magnification). The immunohistochemistry was performed with the two anti-TKTL1 antibodies JFC12T10 and the polyclonal prestige antibody; brown: DAB, counterstain: hematoxiline (blue), magnification x400. Western blot analyses were performed with JFC12T10 (J), USB antibody 1C10 (U) and the SigmaPrestige antibody (P). Marker (M) with molecular weights in kDa. β-actin is shown as loading control.

For protein extraction, 1x10^6^ cells each were lysed in pre-cooled RIPA buffer (Pierce, Rockford, Ilinois) containing phosphatase inhibitors (phosphatase inhibitor cocktails set II, Calbiochem, Germany), proteinase inhibitors (Complete, Roche, Germany) and 2,5 mmol/l Dithiothreitol (DTT) reducing agent (Sigma-Aldrich). The mixture was incubated for 30 min on ice, combined with a thorough shaking every 10 min. Cell lysates were clarified of cell debris by centrifugation at 14,000x g for 5 min through a QIAshredder spin column assembly (Qiagen, Hamburg, Germany) at 4°C. Protein concentration was determined using the Bradford method [44] and coomassie brilliant blue (Roti-Quant; Roth, Karlsruhe, Germany). Afterwards, samples were mixed in 5x loading buffer (Fermentas), denatured at 95°C for 5 min, chilled on ice and stored at −20°C for further analysis. Equal amounts of proteins (20 μg) were loaded on a 10% polyacrylamide gel (SDS-PAGE) and electrophoresed. Proteins were then blotted onto a nitrocellulose membrane (Schleicher & Schuell, Dassel, Germany) for 45 min at 10 V using a semi-dry transfer unit (PeqLab, Erlangen, Germany). The protein transfer was confirmed by reversible membrane staining with Ponceau solution (Sigma-Aldrich). To avoid unspecific binding, the membrane was blocked with 5% non-fat milk (Merck, Darmstadt, Germany) in phosphate buffered saline (PBS)/Tween (0.05%) at room temperature for 1 h. Subsequently, the membrane was incubated with the respective primary anti-TKTL1 antibody at optimal dilution (see below) in PBS/Tween containing 2% non-fat milk at 4°C for 18 h. The following anti-TKTL1 antibodies were used: mouse monoclonal antibody clone JFC12T10 (Linaris GmbH, Wertheim, Germany, stock solution: 1 mg/ml, dilution 1:2,500), polyclonal rabbit Sigma Prestige antibody (HPA000505, Sigma-Aldrich, stock solution: 0.1 mg/ml, dilution 1:1,000), and mouse monoclonal antibody clone 1C10 (US Biological, Biomol GmbH, Hamburg, Germany, stock solution: 1 mg/ml, dilution 1:2,500). After washing with PBS, membranes were incubated with horseradish peroxidase-conjugated secondary antibodies goat-anti-mouse or goat-anti-rabbit, respectively (KPL Gaithersburg, USA, both diluted 1:10,000, stock solution: 0.5 mg/ml) for 60 min at room temperature. A monoclonal mouse anti-β-actin primary antibody, diluted 1:10,000, (Abcam, Cambridge, USA) was used as loading control. Immunoblots were visualized by home-made enhanced chemiluminescence (ECL) consisting of a mixture of 10 ml of solution A (50 mg Luminol (Sigma-Aldrich A4685) dissolved in 200 ml 0,1 mol/l Tris–HCl (pH 8,6)), 1 ml Solution B (11 mg para-hydroxycoumarinacid (Sigma-Aldrich, C9008) dissolved in 10 ml DMSO) and 3 μl of 30% H_2_O_2_[45] with subsequent exposure on an X-ray film (Fuji Super RX medical X-ray films; Fuji Photo Film, Düsseldorf, Germany) for 30 sec (shown in Figure 1) or for 3 min (USB) and 5 min (SigmaPrestige), respectively (shown in Additional file 1: Figure S1).

### RNA extraction and cDNA synthesis

1x10^6^ cells each were disintegrated in Trizol reagent (Invitrogen Life Technologies; Darmstadt, Germany). Total RNA was extracted from Trizol as recommended by the manufacturer. RNA integrity was verified using the Experion automated electrophoresis station (Bio-Rad Laboratories Inc.; Munich, Germany) and the RNA concentration was measured at 260 nm. For first strand cDNA synthesis, 1 μg total RNA was employed using the iScriptcDNA synthesis kit from BioRad. The cDNA synthesis of 1:2 diluted cDNA was performed by heating at 25°C for 5 min, at 42°C for 30 min, and at 85°C for 5 min.

### Reverse Transcriptase (RT) quantitative polymerase chain reaction (qPCR)

Quantitative PCR (qPCR) was performed with MESA Green qPCRMasterMix Kit for SYBR Green containing Meteor Taq hotstart polymerase (Eurogentic GmbH, Köln, Germany). Three sets of **TKTL1** primer pairs were used: **primer pair 1** (150 bp), forward: TAACACCATGACGCCTACTGC, reverse: CATCCTAACAAGCTTTCGCTG [7]**primer pair 2** (80 bp), forward: AAGCCTTTGGGTGGAAC ACTTA, reverse: CTGAGAAGCCTGCCAGAATACC [28]; **primer pair 3** (151 bp), forward: GGTATCTGTTGGTGACGATGGT, reverse: GCACATCCCCTTGGCATTGG [35]. TKTL1 primer pair 1 is located in the non-coding region, and primer pairs 2 and 3 are located in the coding region of TKTL1. The following primer pairs were used as reference genes: **peptidyl propyl isomerase A** (PPIA; access. No. NM_021130.3), forward: TGTCCATGGCAAATGCTGGACCC, reverse: GCGCTCCAT GGCCTCCAC AA (140 bp); **β-actin** (access. No. NM_001101), forward: CCTTGCCATC CTAAAAGCC, reverse: CACGAAAGCAATGCTATCAC (96 bp). qPCR reactions were performed on a CFX96 real-time PCR system (Bio-Rad) operated by CFX Manager Software (version 3.0). The cycler protocol was 5 min at 95°C, 40 cycles of 15 sec at 95°C, 60 sec at 60°C, and 5 min at 72°C. Gene of interest expression was normalized to the reference genes PPIA and β-actin [46]. Fold expression was calculated with the ∆∆Cq method [47]. Post-amplification melting curves were controlled to exclude primer-dimer artifacts and contaminations. Tktl1 mRNA expression was rated positive if the cycle number of the amplification reaction was obviously below the cycle number for HEK293-control transfectants with the three primer pairs. HEK293 cells were demonstrated to be negative for tktl1 mRNA by RT-qPCR and TKTL1 protein by Western blot [21].

### Glucose consumption and lactate production

Cells were seeded at 1x10^5^ cells/ml in 48-well flat bottom tissue plates (TPP; Biochrom) with culture medium and were grown for 24 h. Afterwards, medium was completely replaced, and glucose consumption and lactate production at 21% oxygen were analyzed 24 h later. Cell-free supernatant was snap frozen for analysis and cell numbers were calculated with the crystal violet assay [48]. Frozen supernatants were sent to the central laboratory of the University Hospital of Würzburg, thawed and immediately analyzed for glucose and lactate with the Cobas 8000 modular analyzer series (Roche Diagnostics; Mannheim, Germany). Glucose consumption was calculated as the difference between glucose concentration in cell-free control medium (16.4 ± 0.2 mol/l) (mean ± standard deviation) and glucose concentration remaining in the supernatant of cell cultures after an incubation time of 24 h. Lactate production was calculated as the difference between lactate concentration in the supernatant of cell cultures after an incubation time of 24 h and lactate concentration determined in cell-free control medium (0.29 ± 0.04 mol/l) (mean ± standard deviation). Both results were then correlated to the cell number and displayed as consumption/production per 10,000 cells.

### Paclitaxel and cisplatin sensitivity assay

Adherently growing cell lines were seeded at 5,000 cells per 100 μl of cell culture medium in a 96 well flat bottom tissue culture plate (NUNC Inc., VWR International, Ismaning, Germany) and grown for 24 h prior to treatment with chemotherapeutic drugs. Then, 100 μl cell culture medium containing paclitaxel or cisplatin (both from Sigma-Aldrich) were added, resulting in final drug concentrations ranging from 1% to 200% of the reported peak plasma concentrations achievable in patients [49]. The maximum plasma concentration was given as 15.9 nmol/ml (13.6 μg/ml) for paclitaxel and 12.7 nmol/ml (3.8 μg/ml) for cisplatin [50]. Cells were incubated for further 24 h and cell viability was measured with Cell Counting Kit-8 (CCK-8; Sigma-Aldrich). In brief, supernatant was carefully removed from the cells and a mixture of 100 μl fresh cell culture medium and 10 μl CCK-8 solution were added per well. Cells were incubated for 1 h at 37°C in a humidified incubator before analyzing the substrate reaction at 450 nm in a plate reader (Tecan GENios plus, Tecan Deutschland GmbH, Crailsheim, Germany). All analyses were performed in triplicates. Results were analyzed with the GraphPad Prism software calculating dose–response curves and IC_50_ values.

### Radiosensitivity

Cell lines were seeded at 100 cells in 200 μl cell culture medium in a 96 well flat bottom tissue culture plate (TPP; Biochrom) 24 h prior to radiation. Graded X-irradiation (2, 4, 6 and 8 Gy) was then performed at room temperature using a 6 MV linear accelerator (Primus, Siemens Concord, CA, USA) at a dose rate of 3 Gy/min. Control cells were treated in a similar way, but without irradiation. Each radiation dose was applied in triplicates. 20 min after irradiation cells were cultivated at 37°C in a humidified incubator at 5% CO_2_. Medium was completely replaced after 60 min to start with fresh growth conditions. In addition, cell medium was replaced on day 4 and day 8. Ten days after irradiation, the CCK-8 assay was performed as described above.

## Results

### Detection of TKTL1 protein expression by immunohistochemistry

So far, TKTL1 protein expression was described primarily in paraffin-embedded tumor tissue. Hence, in order to produce comparable datasets, paraffin-embedded cell pellets of all selected cell lines (n = 20) served to analyze the expression of TKTL1 protein by immunohistochemistry. The cell lines assessed in the present study are summarized in Table 1, including tissue origin, supplier and references. In addition, TKTL1-negative human embryonic kidney cells HEK293 have been stably transfected with the expression plasmid pCAG or with a pCAG-based TKTL1 expression plasmid, which resulted in strong expression of the protein, as described elsewhere [21]. All cell lines have been analyzed by immunohistochemistry with the mouse monoclonal antibody clone JFC12T10 and with TKLT1-specific Sigma Prestige (rabbit polyclonal).

Immunohistochemical staining with JFC12T10 and Sigma Prestige antibodies showed a strong cytoplasmatic staining in HEK293-TKTL1 transfectants, with an additional nuclear staining by the Sigma Prestige polyclonal antibody. JFC12T10, but not the Sigma Prestige antibody also stained the cytoplasm of TKTL1-negative HEK293-control cells. The staining intensity of JFC12T10 in HEK293-control and HEK293-TKTL1 transfectants was comparable indicating an unspecific staining pattern of JFC12T10 in HEK293-control transfectants (Figure 1 and Table 2). Cytoplasmic staining of the 17 malign and the three benign cell lines with JFC12T10 ranged from weak (“+”) to very strong (“+++”) (Figure 1, Table 2). Only HT-29 cells remained unstained by JFC12T10. In contrast, cytoplasmic staining intensity with Sigma Prestige polyclonal antibody was faint (“[+]”) in eight cell lines (only PA1 cells were stained “+”), while no staining was observed in the cytoplasm of the remaining eight cell lines (Figure 1, Table 2). With this antibody, no or only weak background staining was observed in benign cells. Both antibodies stained the nuclei of HepG2 cells, and JFC12T10 additionally stained the nuclei of MCF-7, OVCAR, and U87 cells. While the Sigma Prestige polyclonal antibody stained the surface of PA1 and SiHa cells, a surface staining with JFC12T10 was seen in HUVEC and WS1. The most obvious mismatch of staining results from JFC12T10 and the Sigma Prestige antibody was found for HeLa, 23132/87, OVCAR, U251, WiDr and WS1 cells. These six cell lines demonstrated a strong staining by JFC12T10, but no or faint staining by Sigma Prestige antibody (Figure 1, Table 2).

**Table 2:** **Immunohistochemical data**

Cell line	Monoclonal anti-TKTL1 antibody JFC12T10			Polyclonal anti-TKTL1 antibody Sigma Prestige		
	Nucleus	Cytoplasm	Cell surface	Nucleus	Cytoplasm	Cell surface
**control:**						
HEK 293-control	-	++	-	-	-	-
HEK 293-TKTL1	-	+++	-	+	+++	-
**benign:**						
Fibroblasts	-	++	-	-	[+]	-
HUVEC	-	+	++	-	[+]	-
WS1	-	+	++	-	-	-
**malign:**						
HCT116	-	++	-	-	[+]	-
HeLa	-	+++	-	-	-	-
HepG2	[+]	++	-	+	-	-
HT-29	-	-	-	-	-	-
JAR	-	++	-	-	[+]	-
JEG	-	++	-	-	[+]	-
23132/87	-	+	-	-	-	-
MCF-7	[+]	-	-	-	[+]	-
MDA-MB 231	-	++	-	-	-	-
Mel2A	-	++	-	-	[+]	-
OVCAR	+	++	-	-	-	-
PA1	-	+	-	-	+	++
SiHa	-	++	-	-	[+]	+
SKOV3	-	++	-	-	[+]	-
U251	-	+	-	-	-	-
U87	+	+++	-	-	[+]	-
WiDr	-	+++	-	-	-	-

The semiquantitative staining pattern is given for both antibodies (JFC12T10 and Sigma prestige) based on the analysis of two independent staining results per cell line per antibody. Legend: “-“: negative; “[+]”: faint positive; “+”: positive; “++”: strong positive; “+++” very strong positive.

### Detection of TKTL1 protein expression by Western blot analysis

TKTL1 expression was assessed in whole cell lysates of all 20 cell lines and the two control HEK293 cells by Western blot with the monoclonal anti-TKTL1 antibody clones JFC12T10 and 1C10 as well as with the polyclonal Sigma Prestige. All three antibodies detected TKTL1 protein in HEK293-TKTL1 transfectants (Figure 1) with an apparent molecular weight of 65 kDa, which is the expected molecular weight of TKTL1 protein. In contrast to Sigma Prestige and 1C10, JFC12T10 detected additional proteins with molecular weights ranging from 27 kDa to 70 kDa in HEK293-TKTL1 transfectants (Figure 1, Table 3). These additional proteins were also deteced in TKTL1-negative HEK293-control cells and have previously been reported to be insensitive towards transient reduction of TKTL1 expression using specific siRNA oligonucleotides [21]. The most prominent protein had a molecular weight of 27 kDa and was detected in all cell lines, except for WS1 fibroblast cells and primary fibroblasts. Protein bands with an apparent molecular weight of 37, 40, 45, and 55, 70, 100, and >120 kDa were observed in lysates of the majority of cell lines stained with JFC12T10. These protein bands are interpreted as background signals due to the very weak staining even after long exposure (see Additional file 1: Figure S1). Strikingly, Sigma Prestige and 1C10 antibodies did not detect any TKTL1 protein in 15 of 17 malign cell line studied. Monoclonal antibody clone 1C10, which was generated against full-length TKTL1 protein, produced very faint signals with higher and lower molecular weights than expected for TKTL1 in some of the cell lines tested (Figure 1, Table 3). The signal intensity was not increased by an extended exposure time of 3 min. After this very long exposure time, 1C10 detected a very faint signal at the expected molecular weight of TKTL1 in JAR cells. The polyclonal Sigma Prestige antibody, raised against the N-terminus of TKTL1, did not produce any signals except in the positive control HEK293-TKTL1 cells, even after an extended exposure time of 5 min (Additional file 1: Figure S1). To summarize, all three TKTL1-specific antibodies recognize exogenously expressed recombinant TKTL1 protein in HEK293-TKTL1 transfectants. Monoclonal antibody 1C10 did not show any specific signals in the three benign and in 15 out of 17 malign cell lines tested. Polyclonal Sigma Prestige antibody failed to detect TKTL1 in all cell lines tested, indicating a lack of endogenous TKTL1 protein. In contrast, monoclonal antibody JFC12T10 detected numerous additional protein bands with apparent molecular weights above or below the calculated size of TKTL1 (Figure 1 and Table 3). From our data, we cannot exclude the possibility, that the multiple protein signals detected by JFC12T10 antibody, in addition to the full length signal, represent putative smaller TKTL1 fragments derived from the C-terminus, which were not detected by Sigma Prestige and 1C10 antibodies raised against the N-terminus (Sigma Prestige) or the full-length protein (1C10) respectively.

**Table 3:** **Western blot data obtained with the three anti-TKTL1 antibodies JFC12T10 (monoclonal), 1C10 (monoclonal) and Sigma Prestige (polyclonal)**

Cell line	Antibody					
	JFC12T10		1C10		Sigma Prestige	
	Most prominent signal (kDa)	2nd prominent signal (kDa)	Further signals (kDa)	Most prominent signal (kDa)	Further signals (kDa)*	Most prominent signal (kDa)
Fibroblasts	-	-	27/40/55/60	-	-	-
HCT116	27	-	multiple	-	37/55/65	-
HEK 293 -control	27	37	45/55/65/70	-	-	-
HEK 293-TKTL1	65	27/37	multiple	65	-	65
HeLa	27	-	multiple	-	37/40/47/65	-
HepG2	27	37/45	multiple	-	-	-
HT-29	27	-	-	-	-	-
HUVEC	27	37/40/45/50	multiple	-	-	-
JAR	27	37	multiple	-	37/55	-
JEG	27	40	multiple	-	-	-
23132/87	27	37/45	multiple	-	-	-
MCF-7	27	-	multiple	-	37/40/50/70	-
MDA-MB 231	27	40	multiple	-	37/40	-
Mel2A	27	45/40	multiple	-	45/55/70	-
OVCAR	27	42	multiple	-	37/40/50/90	-
PA1	27	37	multiple	-	55	-
SiHa	27	42	multiple	-	-	-
SKOV3	27	37	multiple	-	37740/55	-
U251	27	40/45/55	multiple	-	45	-
U87	27	55	multiple	-	45	-
WiDr	27	42	multiple	-	-	-
WS1	-	-	40/55/70*		40/55/65	-

Western blot staining results with the antibodies JFC12T10, 1C10 and Sigma Prestige of at least two independent blots per cell line per antibody. Legend: “-“ no staining (no signal); “*” very faint staining. The polyclonal Sigma Prestige antibody did not show any signals despite the positive control.

### Detection of *tktl1* mRNA expression by quantitative RT-PCR (RT-qPCR)

Quantitative RT-PCR (RT-qPCR) was performed with three different primer pairs specific for the three *tktl1* mRNA splice variants available in GenBank (PubMed) and published previously [7, 28, 35]. Results of RT-qPCR for all cell lines investigated are shown in Table 4, while Table 5 summarizes basic quantitative PCR data for all three primer pairs. For this, HEK293-TKTL1 transfectants, HEK293 control transfectants and JAR and U251 cells were analyzed. JAR and U251 were identified to weakly express endogenous tktl1 mRNA with all three primer pairs (Table 4). Primer pair 1 (located in the non-coding region of TKTL1 gene) did not recognize coding TKTL1 mRNA in HEK293-TKTL1 transfectants, whereas primer pairs 2 and 3 did. In comparison to JAR and U251 cells, the abundance of tktl1 mRNA in the other malign and benign cells was comparable to or below levels of the TKTL1-negative HEK293 control cell lines, and thus defined as negative (Table 4). The relative expression levels of tktl1 in JAR and U251 were increased up to 560-fold in comparison to the other cells (example for primer pair 3 and JAR and MDA-MB 231).

**Table 4:** **Relative normalized quantification of TKTL1 gene expression with the three published primer pairs TKTL1(1), TKTL1(2) and TKTL1(3)**

Cells	TKTL1(1)		TKTL1(2)		TKTL1(3)	
	Mean ± SD	Cq	Mean ± Stabw	Cq	Mean ± Stabw	Cq
HTC116	0.071 ± 0.063	31.79	7.4x10^−4^ ± 8.0x10^−5^	30.46	3.4x10^−3^ ± 9.4x10^−4^	28.86
HEK 293-TKTL1	0.014 ± 3.1x10^−4^	34.66	48.9 ± 1.14	17.68	48.9 ± 0.80	17.26
HEK 293-control	0.117 ± 0.0046 **^)^	31.63	28.4x10^−4^ ± 2.5x10^−4^ **^)^	28.70	0.010 ± 0.001 **^)^	27.83
HeLa	0.379 ± 0.041	28.40	5.0x10^−5^ ± 2.0x10^−5^	33.13	0.012 ± 0.004	26.04
HepG2	1.3x10^−3^ ± 9.2x10^−4^	36.75	2.0x10^−5^ ± 3.0x10^−5^	35.03	1.1x10^−4^ ± 2.0x10^−5^	33.02
HT-29	2.5x10^−3^ ± 1.6x10^−3^	35.36	2.0x10^−5^ ± 2.0x10^−5^	33.83	4.0x10^−5^ ± 3.0x10^−5^	33.84
HUVEC	0.194 ± 0.05	32.23	2.4x10^−4^ ± 2.3x10^−4^	33.72	1.8x10^−4^ ± 3.0x10^−5^	34.99
JAR	0.462 ± 0.09	25.64	0.037 ± 0.0031	25.15	0.028 ± 8.30x10^−4^	24.08
JEG	0.039 ± 0.006	30.12	1.6x10^−4^ ± 4.0x10^−5^	30.90	3.6x10^−4^ ± 3.0x10^−5^	29.58
23132/87	9.9x10^−3^ ± 0.014	34.13	9.0x10^−5^ ± 9.0x10^−5^	32.81	1.6x10^−4^ ± 1.0x10^−5^	32.79
MCF-7	2.4x10^−4^ ± 0.002	34.76	1.0x10^−5^ ± 1.0x10^−5^	36.85	1.0x10^−5^ ± 1.0x10^−5^	35.64
MDA-MB 231	3.5x10^−4^ ± 3.3x10^−4^	37.34	1.0x10^−5^ ± 1.0x10^−5^	33.87	5.0x10^−5^ ± 1.0x10^−5^	32.79
Mel2a	0.161 ± 0.012	30.81	1.6x10^−3^ ± 3.4x10^−4^	28.52	4.4x10^−3^ ± 2.3x10^−4^	28.69
OVCAR	0.333 ± 0.073	28.71	5.0x10^−5^ ± 6.0x10^−5^	33.51	1.7x10^−4^ ± 5.0x10^−5^	32.33
PA1	0.021 ± 0.007	33.36	5.1x10^−4^ ± 1.0x10^−4^	30.83	1.3x10^−3^ ± 1.4x10^−4^	30.06
SiHa	0.023 ± 0.020	32.33	1.0x10^−5^ ± 1.0x10^−5^	35.57	4.0x10^−5^ ± 4.0x10^−5^	34.18
SKOV3	0.084 ± 0.062	31.16	5.1x10^−4^ ± 1.4x10^−4^	30.43	1.2x10^−3^ ± 3.6x10^−4^	29.99
U251	1.000 ± 0.117	22.84	0.046 ± 0.0015	23.16	0.036 ± 9.7x10^−4^	22.06
U87	1.4x10^−3^ ± 2.8x10^−4^	36.81	1.0x10^−5^ ± 1.0x10^−5^	35.97	7.0x10^−5^ ± 1.0x10^−4^	33.84
WiDr	3.6x10^−3^ ± 2.9x10^−4^	34.95	2.0x10^−5^ ± 3.0x10^−5^	34.14	8.0x10^−5^ ± 2.0x10^−4^	33.13
WS1	0.010 ± 0.007	35.56	1.0x10^−5^ ± 1.0x10^−5^	38.50	6.0x10^−5^ ± 4.0x10^−5^	35.59

For the quantification of TKTL1 gene expression, actin and PPIA were used as endogenous controls. In addition to values of relative normalized expression the cycle of quantification (Cq) for each PCR amplicon is indicated (Cq presents the cycle number at which the amount of amplified PCR amplicon reaches the threshold level). Cq values higher than the cut-off of 35 was not considered as a reliable Cq expression value. It is obvious, that the relative expression levels of TKTL1 in most tested malign and benign cell lines are decreased in comparison to JAR and U251. For HEK293-TKTL1 transfectants, the PCR amplicons amplified with primer pairs 2 and 3 were successfully proofed for TKTL1 sequence, whereas for HEK293-control transfectants, amplicons amplified with the three primer pairs were without TKTL1 sequence (data not shown).

**Table 5:** **Comparison of PCR efficiency for the TKTL1 gene obtained with the three primer pairs**

Cell line	Primer pairs	E (%)	Slope	R ^2^	Cq (1:5)	Cq (1:320)	Cq (NTC)
**HEK293-TKTL1**	TKTL1(1)	----	-----	----	35.38 ± 0.78	35.95 ± 0.58	>40
	TKTL1(2)	95.7	−3.429	0.991	22.09 ± 0.04	28.29 ± 0.46	>38
	TKTL1(3)	94.9	−3.451	0.997	22.05 ± 0.24	28.14 ± 0.20	>40
	PPIA	94.2	−3.470	0.994	22.78 ± 0.25	29.15 ± 0.10	>38
	β-actin	95.1	−3.445	0.999	19.63 ± 0.03	25.89 ± 0.02	>32
**HEK293-control**	TKTL1(1)	-----	-----	-----	33.74 ± 1.19	35.89 ± 0.85	>37
	TKTL1(2)	-----	-----	-----	27.78 ± 0.03	27.98 ± 0.02	>37
	TKTL1(3)	-----	-----	-----	30.33 ± 0.51	33.87 ± 1.01	>35
	PPIA	90.2	−3.581	0.999	17.39 ± 0.05	23.94 ± 0.08	>35
	β-actin	91.1	−3.554	1.000	17.38 ± 0.08	23.79 ± 0.06	>31
**U251**	TKTL1(1)	100.3	−3.315	0.987	26.44 ± 0.32	32.49 ± 0.10	>39
	TKTL1(2)	103.5	−3.242	0.997	27.78 ± 0.02	33.69 ± 0.42	>36
	TKTL1(3)	103.3	−3.246	0.989	26.69 ± 0.10	32.88 ± 0.04	>36
	PPIA	99.3	−3.339	0.996	18.92 ± 0.07	25.08 ± 0.07	>37
	β-actin	99.3	−3.339	1.000	15.53 ± 0.02	21.57 ± 0.02	>31
**JAR**	TKTL1(1)	94.3	−3.467	0.987	27.67 ± 0.567	33.93 ± 0.421	>40
	TKTL1(2)	92.1	−3528	0.974	28.64 ± 0.26	35.42 ± 0.53	>36
	TKTL1(3)	90.1	−3.584	0.938	27.91 ± 064	34.48 ± 0.56	>37
	PPIA	93.5	−3.489	0.995	14.82 ± 0.01	24.99 ± 0.05	>40
	β-actin	98.1	−3.367	0.999	14.82 ± 0.01	20.96 ± 0.02	>31

The results are shown for the HEK293-TKTL1 and HEK293-control transfectants as well as for the cell lines JAR and U251 with the highest expression levels. The following information about the performance of RT-qPCR for the three TKTL1 primer pairs as well as for the primer pairs for reference (housekeeping) genes β-actin and PPIA is given: the PCR efficiency (E in %) and the slope of the standard curves (a PCR efficiency of 100% corresponds to a slope of −3.32 and a slope of less −3.32 is indicative of a PCR efficiency <100%), the correlation coefficient R^2^ reflecting the linearity of the standard curve (ideally, R^2^ = 0.999) and the quantification (threshold) cycles Cq for cDNA dilutions 1:5 and 1:320. In addition, Cq values for no template controls (NTC) are shown. The amplification efficiencies of TKTL1 gene and the endogenous references genes β-actin and PPIA are comparable. It is worth to mention that the performance of PCR with the three primer pairs did not demonstrate a dynamic range for the enzyme reaction allowing to construct a standard curve (copy number versus Cq value), and therefore to calculate the efficiency of the enzyme reaction.

### Glucose consumption and lactic acid formation

**Figure 2:**
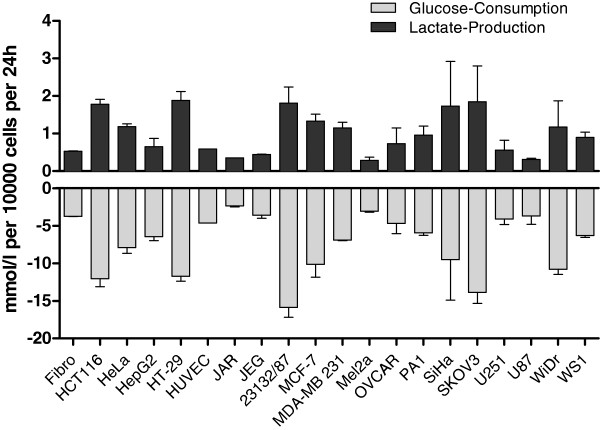
**Glucose consumption and lactic acid formation.** Glucose consumption (grey bars) and lactate production (black bars) of the cell lines *in vitro* were determined as described in material and methods and are shown for 21% oxygen. Glucose consumption and lactate production are calculated for 10,000 cells after 24 h in culture.

High expression of TKTL1 was put into context of enhanced glucose consumption within the pentose-phosphate pathway, resulting in increased lactic acid production [7]. In order to correlate both parameters with TKTL1 expression, glucose usage and lactic acid production were analyzed for confluent cell cultures after 24 h of culture at 21% oxygen. Data are summarized in Additional file 2: Table S1. Mean glucose consumption was 7.4 mmol/l per 10,000 cells (range: 1.9-21.4 mmol/l) after 24 h across all cell lines tested (n = 20) (Figure 2, light grey columns). The corresponding lactic acid production had a mean value of 1.03 mmol/l per 10,000 cells (range: 0.2-4.1 mmol/l) after 24 h (Figure 2, dark grey columns). All cell lines including benign cells showed lactic acid production 21% oxygen, thus demonstrating the “Warburg effect”. Glucose consumption and lactate production were correlated as expected. HCT116, HT-29, 23132/87, SiHa, and SKOV3 cells displayed the highest levels of glucose consumption and lactate production, whereas fibroblasts, HepG2, HUVEC, JEG, JAR, Mel2a, U251, and U87 displayed the lowest levels (Figure 2). JAR and U251 revealed to be the only cell lines within the panel analyzed in the present study to express TKTL1. Both cell lines did not demonstrate an outstanding glucose consumption and production of lactic acid.

### Effects of paclitaxel, cisplatin and radiation on cell viability

**Figure 3:**
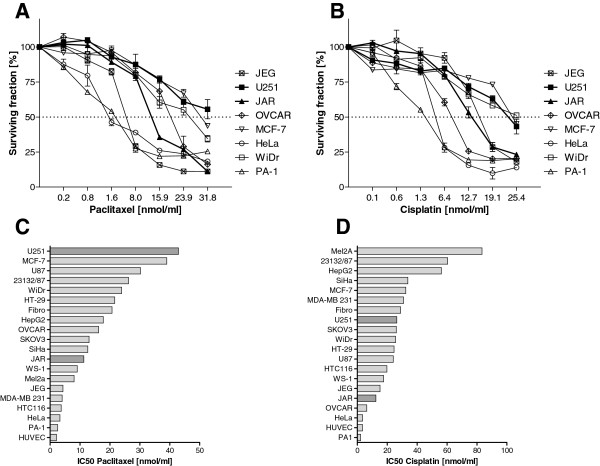
**Effects of paclitaxel and cisplatin on cell viability.** Representative dose–response curves of selected cell lines in response to paclitaxel (**A**) and cisplatin (**B**). Paclitaxel was used in concentrations ranging between 0.2 and 31.8 nmol/ml, and cisplatin was used in concentrations ranging between 0.1 and 25.4 nmol/ml. Cell lines are arranged according to increasing IC_50_ values, from sensitive to resistant cell lines, for paclitaxel (**C**) and cisplatin (**D**). Cell lines found positive for tktl1 mRNA are highlighted.

**Figure 4:**
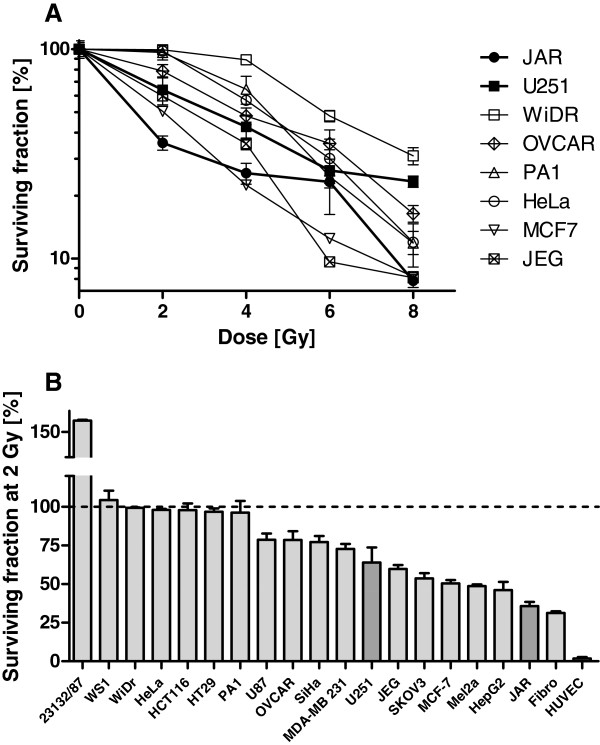
**Effect of radiation on cell viability.** (**A**) Representative dose–response curves of selected cell lines in response to radiation with doses between 2 Gy and 8 Gy. (**B**) Cell lines are arranged according to decreasing cell viability after radiation with 2 Gy. This dose lead to survival of a small number (2 ± 1%) of radiated HUVEC control cells in contrast to other tested doses where no HUVEC cells survived (not shown). Cell lines found positive for tktl1 mRNA are highlighted.

Paclitaxel and cisplatin are widely used chemotherapeutic drugs for clinical treatment of solid cancers and are suitable for sensitivity tests. Therefore, all selected cell lines (n = 20) were subjected to treatment with paclitaxel and cisplatin in concentrations ranging from 0.2-31.8 nmol/ml and 0.1-25.4 nmol/ml, respectively. Representative results of dose–response curves after treatment with paclitaxel and cisplatin are shown (Figure 3A, 3B). From the resulting survival curves, IC_50_ values were calculated (Figure 3C, 3D). HUVEC, HeLa and PA-1 cells were highly sensitive to paclitaxel and cisplatin (IC_50_ < 5 nmol/ml), whereas U251 and MCF-7 were resistant to paclitaxel and Mel2A, and 23132/87 and HepG2 were resistant to cisplatin (IC_50_ > 35 nmol/ml). JAR cells displayed intermediate and high sensitivity towards placlitaxel and cisplatin, respectively (Figure 3). Thus, the investigated cell lines exhibited a broad range of resistance and sensitivity to paclitaxel and cisplatin.Next, all selected cell lines were irradiated at doses ranging from 2 to 8 Gy and survival was determined. Representative dose–response curves for radiation are shown for JAR, U251, WiDr, OVCAR, PA1, HeLa, MCF7, and JEG cell lines in Figure 4A. Radiosensitivity was derived from the surviving fraction of cells at 2 Gy. This dose was effective at killing the majority of irradiated HUVEC (surviving fraction 2 ± 1%), and larger doses led to complete eradication of HUVEC (data not shown). All cell lines tested (n = 20) demonstrated a broad range of radiosensitivity. The stomach cancer cell line 23132/87 was highly resistant to radiation and grew to a surviving fraction of 163 ± 1.8% (Figure 4B). WS1, WiDr, HeLa, HCT116, HT29, and PA1 cells displayed strong resistance to radiation, with a survival fraction at 2 Gy of approximately 100% (Figure 4B). In contrast, HepG2, JAR, fibroblasts and HUVEC cells were characterized by radiosensitivity, with survival fractions below 50%. U251 cells revealed intermediate sensitivity towards radiation, with a survival percentage (2Gy) of 64 ± 16%. In summary, the tested cell lines demonstrated broadly varying degrees of radiosensitivity.

### TKTL1 expression and relation to chemo- and radioresistance as well as to the “Warburg effect” *in vitro*

Performing RT-qPCR with three different primer pairs, we identified two of 17 malign human cell lines (12%) characterized by weak endogenous tktl1 mRNA expression (JAR and U251). Immunohistochemical proof of TKTL1 protein in cytoplasm, the expected location of TKTL1 as a protein which is supposed to be instrumental in the pentose-phosphate pathway, was successful in these two cell lines with JFC12T10. A very faint staining was seen for JAR cells while U251 cells were negative using Sigma Prestige polyclonal antibody, thus confirming negative Western blot results. All other cell lines demonstrated much lower tktl1 mRNA expression levels and no detection of TKTL1 protein in cell lysates by anti-TKTL1 antibodies 1C10 and Sigma Prestige, but multiple detections of proteins with apparent molecular weights above or below the calculated size of TKTL1 by anti-TKTL1 antibody JFC12T10. A relation of glucose consumption, lactic acid production and chemo- and radioresistance with expression levels of TKTL1 was not observed in any cell line tested in the present study. JAR and U251 cells did not show outstanding “aggressiveness” as measured by their resistance to both chemotherapeutic drugs and radiation or their ability to produce lactate (Figures 2, 3 and 4). While U251 was resistant to paclitaxel treatment and moderately sensitive to cisplatin, JAR cells were sensitive to both chemotherapeutic drugs (Figure 3). In comparison to most other cells tested, both cell lines were sensitive to radiation (Figure 4) and showed a moderate Warburg effect (Figure 2). Therefore, the proposed link between TKTL1 expression and chemo- and/or radioresistance as well as increased lactic acid formation by tumor cell lines was not confirmed by our results. In fact, TKTL1 expression was generally rare among the cell lines tested herein.

## Discussion

Transketolase-like 1 (TKTL1) has gained increased attention due to several studies showing a TKTL1-positive staining of malignant tumor tissue of different origins [15, 16, 20, 22–24, 35, 40, 41, 43, 51] and its subsequent designation as a “proto-oncogene” [9]. Furthermore, several cancer cell lines have been reported to be positive for TKTL1 protein upon Western blot analysis [7, 21, 29, 52–54], cyto-immunohistochemistry [50, 55] and RT-qPCR. For example, Sun and coworkers analyzed TKTL1 expression in six HNSCC cell lines via Western blot with the JFC12T10 antibody and found that TKTL1 was relatively overexpressed in two cell lines (FaDU and UM22B) compared with levels in normal mucosal samples [29].

In this study, we tested three commercially available anti-TKTL1 antibodies for Western blot and immunohistochemistry staining as well as three primer pairs used in previously published work for the detection of tktl1 mRNA by RT-qPCR [7, 28, 35]. Two independent antibody batches of each antibody were tested with identical results in Western blot and immunohistochemistry experiments. The reliability and specificity of each antibody and primer pair was addressed and thoroughly quantified using HEK293-TKTL1 transfectants producing full-length TKTL1 and their TKTL1-negative counterparts HEK293-control cells [21]. Assessment of tktl1 mRNA expression disclosed a strong expression of the transgene in HEK293-TKTL1 cells. Amongst all other cancer cell lines tested, only JAR and U251 cells exhibited an – albeit very weak - expression, with the remaining cell lines expressing levels of tktl1 transcripts comparable or even lower than the TKTL1-negative HEK293 controls. The discrepant results for RT-qPCR and protein expression described by us for several cell lines used in this study were also observed by Benz et al. for cell lines tested by them (Figure 1a + b in [52]). For example, they found a TKTL1 signal in SW620 cells with Western blot using the JFC12T10 antibody, but no signal with RT-qPCR. Vice versa, SW948 cells are positive for tktl1 mRNA but negative for TKTL1 protein [52]. In contrast, Kayser at al. discuss a tight correlation of immunohistochemically detected TKTL1 expression using JFC12T10 and mRNA despite “doubts of the specificity of the antibody clone JFC12T10” [20] but they do not present results for this statement.

HeLa cells [56], colorectal carcinoma cell line HCT-116 [28] and other carcinoma cell lines [41, 52] have been reported to express tktl1 mRNA. In contradiction, Hartmannsberger et al. noted the absence of endogenous tktl1 mRNA in a panel of different tumor cells including HeLa and MCF-7 [21], which is consistent with our results and was further corroborated by results from Mayer et al. [54]. However, by using a primer pair identical to our primer pair 1 located in the non-coding region in a standard 40 cycles RT-PCR, Chen et al. found tktl1 mRNA in HeLa cells [56]. Bentz and coworkers identified WiDr cells as negative for tktl1 mRNA [52] in accordance with our results. However, in contrast to our study, they observed high expression of tktl1 mRNA in HT-29 cells [52]. Discrepancies between our and other RT-qPCR and PCR data could be due to either different expression profiles of the cell lines in different laboratories, (un)authenticated cell lines or varying subclones [57, 58]. In summary, our results suggest that tktl1 mRNA expression is a rare phenomenon in a broad panel of malign and benign cell lines *in vitro*.

Interestingly, the vast majority of studies based on antibody-based techniques published so far used the commercially available mouse monoclonal IgG2_b_ anti-TKTL1 antibody clone JFC12T10, first described in 2005 [7]. JFC12T10 was generated against a 22 kDA C-terminal fragment of a recombinant TKTL1 protein. JFC12T10 stained a histidine-tagged full length TKTL1 protein expressed in *E. coli* with putative smaller cleavage products. Furthermore, JFC12T10 showed a unique expression pattern of five different tumor cell lines with a predominant protein band at 75 kDa by Western blot analysis [7]. Indeed, the cell lines investigated in the present study showed a faintly stained protein band at approximately 75 kDa, among numerous other protein bands. However, for most of the cells, JFC12T10 detected approximately 10 different proteins with a predominant band at approximately 27 kDa. This staining pattern was comparable to results obtained by using JFC12T10 in HEK293-control transfectants, which are devoid of TKTL1 protein [21]. This indicates multiple unspecific binding targets recognized by JFC12T10 in a broad variety of cell lines, including the HEK293-TKTL1 transfectants. In the latter, a strong additional signal around 65 kDa representing the calculated size of full-length recombinant TKTL1 protein [7] was detected, which was also described by Bentz and coworkers previously [52].

The second anti-TKTL1 antibody used for Western blot analysis was the monoclonal antibody clone 1C10 that detected a distinct single TKTL1 protein with approximately 65 kDa in HEK293-TKTL1 transfectants, without the additional protein bands seen with JFC12T10. Also, polyclonal Sigma Prestige antibody did not recognize additional proteins with different molecular weights in any of the cell lines. The marginal signals in a few cell lines seen with monoclonal 1C10 antibody most probably represents unspecific background, which was eventually similar in quality but inferior in quantity as compared to JFC12T10. Thus, monoclonal 1C10 and polyclonal Sigma Prestige antibody detect exogenously expressed TKTL1 in a highly specific manner and as a protein of expectedly 65.4 kDa.

However, using a rabbit polyclonal antibody (Gene Tex), Li et al. detected endogenous TKTL1 expression in MCF-7 and HeLa cells by Western blot [53]. This result is in contrast to our data, since none of the three tested antibodies recognized TKTL1 protein in those cell lines. This might reflect the different specificity of antibodies and/or cell batches used. Likewise and in discordance to our findings, Bentz et al. described cell line HT29 as highly positive for TKTL1 protein expression in Western blotting with antibody JFC12T10 [52]. Of note, Bentz et al. used JFC12T10 in a remarkably high concentration (diluted 1:100 compared to 1:2500 as in the present work) and did not present any information about the source of the cell line. Thus, the difference in the results of Bentz as well as of Li to our findings despite using the same cell lines underline the importance for an interlaboratory cell identification screening program [57, 58]. It is worth to note, that numerous publications about TKTL1 displayed small clippings of Western blot data only and in some cases even the molecular weight markers were omitted [11, 28, 53] or only one molecular weight marker was given at 75 kDa [59]. These depictions complicate the appraisal of results.

Similarly, immunohistochemistry analyses presented here support a rather rare expression of TKTL1 protein in cancer and normal cell lines. In 2 out of 3 benign and 8 out of 17 malign cell lines tested in the present study, TKTL1 protein was detectable in the cytoplasm with the two antibodies JFC12T10 and SigmaPrestige polyclonal antibody by immunohistochemistry. For only one of these positively stained cell lines (JAR) a weak expression of tktl1 mRNA was detectable. Therefore JAR appears to be positive for endogenous TKTL1 expression by RT-qPCR and immunohistochemistry, however failed to be detected by Western blot. The other cell line expressing tktl1 mRNA, U251, was positive in immunohistochemistry with the JFC12T10 antibody only and also showed no specific signal in Western blot.

In summary, two cell lines (JAR, and U251) were found positive for both endogenous mRNA expression as well as TKTL1 protein by immunohistochemistry with one or two different anti-TKTL1 antibodies. Intriguingly, these cell lines did neither exhibit an extraordinary production of lactic acid at 21% oxygen *in vitro* (Warburg effect) nor greater resistance against taxane and cisplatin or ionizing radiation than cells without clear TKTL1 detection. These findings are inconsistent with the hypothesis of TKTL1 being a predictor of an increased “Warburg effect” and special robustness of tumor cells against chemotherapy and radiation *in vitro*.

## Conclusions

In this study we have demonstrated the necessity to carefully prove TKTL1 expression on mRNA and protein levels with multiple analytic tools (antibodies, primer pairs) to identify cells with robust endogenous TKTL1 expression *in vitro*. TKTL1 expression emerged as a rather rare event in cultured cell lines. The two out of 17 human cancer cell lines JAR and U251, positive for tktl1 mRNA and TKTL1 protein by immunohistochemistry and Western blot for at least one antibody showed neither an outstanding production of lactic acid nor increased resistance against the chemotherapeutic drugs paclitaxel and cisplatin or to ionizing radiation, respectively, compared to the other cell lines.

## Electronic supplementary material

Additional file 1: Figure S1: Long time exposure of Western Blot. To make the WB results comparable, we exposed the x-Ray film to an extent, that the positive control cells (HEK293-TKTL1) resulted in a comparable intensity of band signal at 65.4 kDa. The negative HEK293 control cells as well as the tktl1 mRNA positive cell lines JAR and U251 were also shown. (PPT 10 MB)

Additional file 2: Table S1: Concentrations of glucose and lactate. Mean and standard deviation is shown for glucose consumption and lactate production of cell lines analyzed after 24 h of culture calculated per 10^5^ cells each. Summary of three independent experiments. (DOC 40 KB)

## Abbreviations

ATP: Adenosine triphosphate
EC number: Enzyme Commission number
PPP: Pentose phosphate pathway
RT-qPCR: Reverse transcriptase (RT) quantitative polymerase chain reaction (qPCR)
TKT: Transketolase
TKTL1: Transketolase like enzyme 1.
