# Supplementary material for: TKTL1 expression in human malign and benign cell lines
Source: BMC Cancer. 2015 Jun 10;15:2. doi: 10.1186/1471-2407-15-2 (PMC4506423; doi:10.1186/1471-2407-15-2)
Supplement: Supplementary file 1 — Additional file 1: Figure S1: Long time exposure of Western Blot. To make the WB results comparable, we exposed the x-Ray film to an extent, that the positive control cells (HEK293-TKTL1) resulted in a comparable intensity of band signal at 65.4 kDa. The negative HEK293 control cells as well as the tktl1 mRNA positive cell lines JAR and U251 were also shown. (PPT 10 MB) [file 12885_2014_5248_MOESM1_ESM.ppt]

## Slide 1
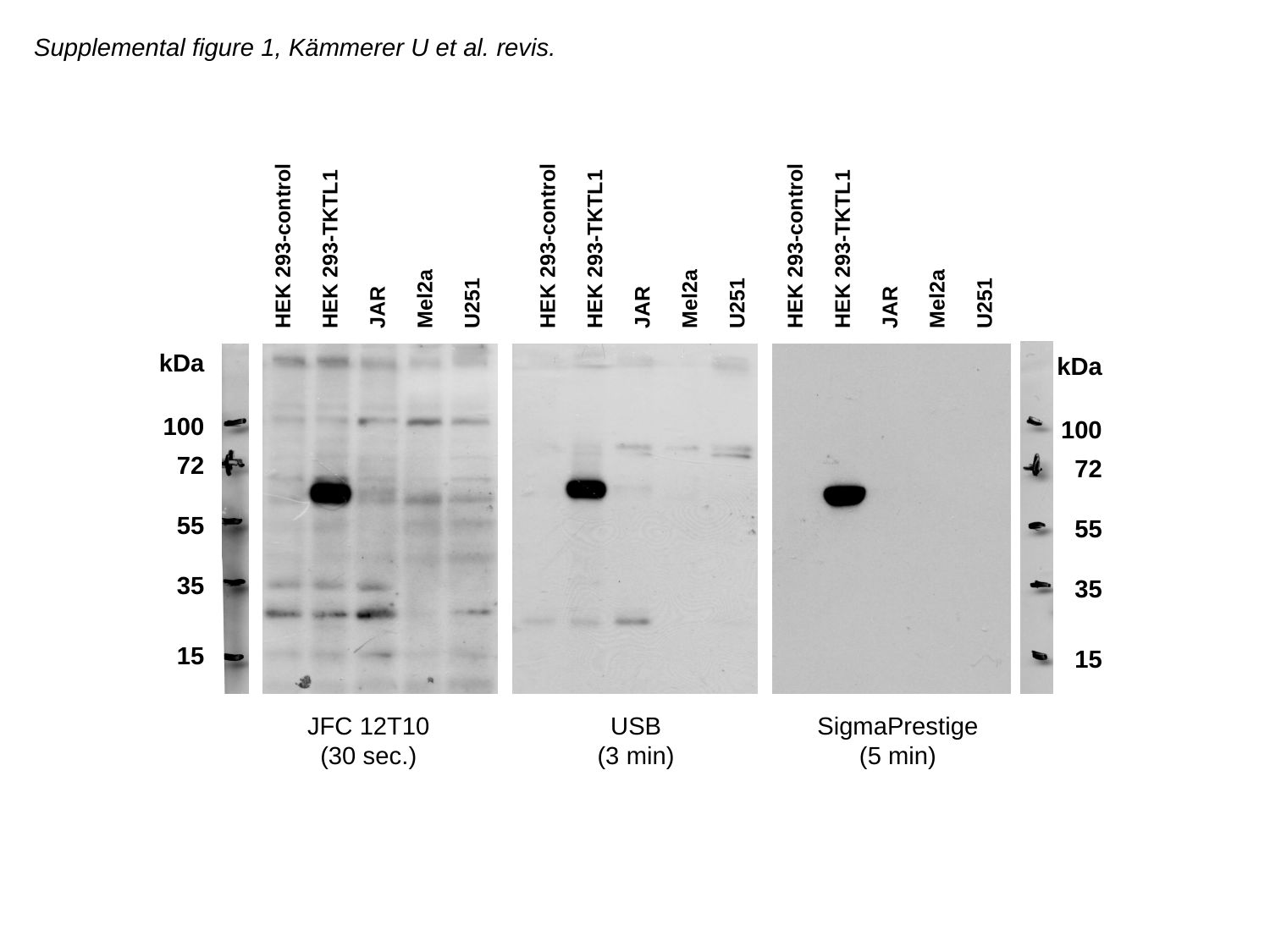

Supplemental figure 1, Kämmerer U et al. revis.
HEK 293-control
HEK 293-TKTL1
Mel2a
U251
JAR
HEK 293-control
HEK 293-TKTL1
Mel2a
U251
JAR
HEK 293-control
HEK 293-TKTL1
Mel2a
U251
JAR
kDa
100
72
55
35
15
kDa
100
72
55
35
15
JFC 12T10
(30 sec.)
USB
(3 min)
SigmaPrestige
(5 min)
