# Supplementary material for: TKTL1 expression in human malign and benign cell lines
Source: BMC Cancer. 2015 Jun 10;15:2. doi: 10.1186/1471-2407-15-2 (PMC4506423; doi:10.1186/1471-2407-15-2)
Supplement: Supplementary file 2 — Additional file 2: Table S1: Concentrations of glucose and lactate. Mean and standard deviation is shown for glucose consumption and lactate production of cell lines analyzed after 24 h of culture calculated per 105 cells each. Summary of three independent experiments. (DOC 40 KB) [file 12885_2014_5248_MOESM2_ESM.doc]

Additional file 1: Table S1: Glucose consumption and lactate production of

cell lines analyzed after 24 h of culture

| Cells | Glucose consumption  mmol/l per 10^5^ cells  per 24 h | Lactate production  mmol/l per 10^5^ cells  per 24 h |
| --- | --- | --- |
|  | **Mean ± SD** | **Mean ± SD** |
| **Fibroblasts** | **-3.7 ± 0.1** | **0.5 ± 0.1** |
| **HTC116** | **-12.1 ± 1.5** | **1.8 ± 0.2** |
| **HeLa** | **-7.9 ± 1.1** | **1.2 ± 0.1** |
| **HepG2** | **-6.5 ± 0.7** | **0.7 ± 0.3** |
| **HT-29** | **-11.7 ± 0.9** | **1.9 ± 0.3** |
| **HUVEC** | **-4.6 ± 0.1** | **0.6 ± 0.02** |
| **JAR** | **-2.4 ± 0.2** | **0.4 ± 0.01** |
| **JEG** | **-3.6 ± 0.5** | **0.4 ± 0.01** |
| **23132/87** | **-15.9 ± 1.5** | **1.8 ± 0.6** |
| **MCF-7** | **-10.1 ± 2.4** | **1.3 ± 0.3** |
| **MDA-MB 231** | **-6.9 ± 0.1** | **1.2 ± 0.2** |
| **Mel2a** | **-3.1 ± 0.2** | **0.3 ± 0.1** |
| **OVCAR** | **-4.7 ± 2.0** | **0.3 ± 0.1** |
| **PA1** | **-5.9 ± 0.5** | **1.0 ± 0.3** |
| **SiHa** | **-9.5 ± 7.6** | **1.7 ± 0.5** |
| **SKOV3** | **-13.9 ± 2.1** | **1.8 ± 1.4** |
| **U251** | **-4.1 ± 1.0** | **0.6 ± 0.4** |
| **U87** | **-3.7 ± 1.5** | **1.2 ± 1.0** |
| **WiDr** | **-10.8 ± 1.0** | **1.2 ± 1.0** |
| **WS1** | **-6.3 ± 0.4** | **0.9 ± 0.2** |
